# Electroacupuncture Mitigates Hippocampal Cognitive Impairments by Reducing BACE1 Deposition and Activating PKA in APP/PS1 Double Transgenic Mice

**DOI:** 10.1155/2019/2823679

**Published:** 2019-05-15

**Authors:** Yinshan Tang, Shujun Shao, Yu Guo, You Zhou, Jin Cao, Anping Xu, Jihong Wu, Zhigang Li, Dulian Xiang

**Affiliations:** ^1^Department of Rehabilitation and Traditional Chinese Medicine, The Second Affiliated Hospital of Zhejiang University School of Medicine, Hangzhou, Zhejiang 310009, China; ^2^School of Acupuncture-Moxibustion and Tuina, Beijing University of Chinese Medicine, Beijing 100029, China; ^3^Department of Psychiatry, Massachusetts General Hospital, Harvard Medical School, Massachusetts 02129, USA

## Abstract

Increased amyloid-*β* (A*β*) plaque deposition is thought to be the main cause of Alzheimer's disease (AD). *β*-Site amyloid precursor protein cleaving enzyme 1 (BACE1) is the key protein involved in A*β* peptide generation. Excessive expression of BACE1 might cause overproduction of neurotoxins in the central nervous system. Previous studies indicated that BACE1 initially cleaves the amyloid precursor protein (APP) and may subsequently interfere with physiological functions of proteins such as PKA, which is recognized to be closely associated with long-term potentiation (LTP) level and can effectively ameliorate cognitive impairments. Therefore, revealing the underlying mechanism of BACE1 in the pathogenesis of AD might have a significant impact on the future development of therapeutic agents targeting dementia. This study examined the effects of electroacupuncture (EA) stimulation on BACE1, APP, and p-PKA protein levels in hippocampal tissue samples. Memory and learning abilities were assessed using the Morris water maze test after EA intervention. Immunofluorescence, immunohistochemistry, and western blot were employed to assess the distribution patterns and expression levels of BACE1, APP, and p-PKA, respectively. The results showed the downregulation of BACE1 and APP and the activation of PKA by EA. In summary, EA treatment might reduce BACE1 deposition in APP/PS1 transgenic mice and regulate PKA and its associated substrates, such as LTP to change memory and learning abilities.

## 1. Introduction

AD is a nonreversible and deleterious neurodegenerative disease and the most common cause of dementia worldwide, accounting for 60-80% of these diseases [1]. The pathogenesis of AD manifests as cognitive impairments, personality change, and behavioral abnormalities, which ultimately lead to death [2, 3]. Increasing prevalence of AD poses a major threat to the elderly population worldwide, especially in developing countries [4]. Additionally, socioeconomic costs of long-term treatment and hospital care have increased annually, creating burgeoning obstacles in social development [2]. Hence, there is an urgent need for further exploring the underlying mechanism of AD and developing effective treatments for dementia.

The brains of AD patients are characterized by pathological features, including amyloid-*β* (A*β*) senile plaques and neurofibrillary tangles, which lead to damage and atrophy to neuronal structures. The accumulated, aggregated, or uncleared A*β* peptides, forming both soluble A*β* oligomers and insoluble amyloid plaques, are crucial for the occurrence and deterioration of AD [5]. Moreover, A*β* peptides in the form of amyloid plaques are directly or indirectly responsible for the ensuing neurodegeneration and memory loss. Numerous investigations have been conducted to explain the accumulation of A*β* [6, 7]. *α*-, *β*-, and *γ*-secretases are considered to be the main enzymes that cause proteolytic cleavage of APP and produce A*β* peptides [8–10]. Accumulative evidence implicated that A*β* secretion resulted from *α*- and *β*-secretase cleavage of APP, respectively, to induce sAPP-*α* and sAPP-*β* [7, 11, 12]. However, sAPP-*α* has low expression and derives from the nonamyloidogenic pathway. Its effects differ from sAPP-*β*, which is identified as a neurodegenerative factor. Hence, *β*-secretase is considered as the first step in the generation of A*β* peptides and initiates the amyloid cascade [13], producing sAPP-*β* fragment and a C-99 C-terminal membrane [14]. Subsequently, cleavage of C-99 by *γ*-secretase complex including presenilin leads to the release of the AD-associated A*β* fragment [11, 15]. Accordingly, BACE1 is identified as the *β*-secretase [16], which acts as a key enzyme that triggers the production of A*β* in both cell lines and transgenic mice. BACE1 levels are upregulated under stress and oxidative stress [17, 18], which are associated with the increasing incidence of AD. Over the last decade, numerous BACE1 inhibitors have been tested in clinical trials. The administration of BACE1 inhibitor could dramatically decrease the A*β* level and attenuate behavioral deficits in APP transgenic mice [12, 19], yet their brains naturally possess high concentrations of soluble A*β*. The deletion of BACE1 gene decreased A*β* secretion and improved learning and memory in AD mouse models [20].

Protein kinase A (PKA), a predominant positive modulator of hippocampal LTP, is reported to indispensably participate in the efficacy of hippocampus-based memory [21]. Cyclic adenosine monophosphate (cAMP) is an essential upstream mediator that regulates diverse types of cell-specific processes; intracellular cAMP directly regulates the expression of the signaling protein PKA. The activation of cAMP-PKA signaling is essential to enhance synapse plasticity through upregulating the release of synaptic vesicle in presynaptic terminal [22]. By stimulating the presynaptic cell, LTP could be induced, which would in turn maintain the upper stable state of synapse consolidation [23]. Over the years, an emerging crucial memory player cAMP response element-binding protein (CREB) is one of the best-studied transcription factors implicated in the hippocampus-based memory formation process, and its disruption causes cognitive disabilities. The PKA/CREB signaling pathway is critical for the retrieval of impaired memory [24, 25]. PKA acts as the upstream regulator of CREB and is essentially involved in the activation/phosphorylation of CREB. Higher expression of PKA would potentially improve synaptic plasticity via a PKA-CREB-mediated pathway [25]. In addition, the protein scaffolding family of A-kinase-anchoring proteins (AKAPs) helps in regulating the localization and compartmentalization of PKA that regulates the activation of postsynaptic multiple proteins. One possibility is that AKAPs mediate postsynaptic plasticity via regulating the release of synaptic vesicle and activate PKA phosphorylation function [26, 27]. Thus, the activation of anchored PKA affects the regulation of localized cAMP signaling pathway events [28]. Moreover, PKA recognizes and targets its substrates via AKAPs; anchored PKA critically regulates the activity of synaptic plasticity during memory storage [26]. Recent studies suggested that an elevated BACE1 protein level reduces PKA expression in the mouse brain. However, these effects of BACE1 are independent of BACE1 enzymatic activity and A*β* levels [29].

Acupuncture has been widely applied in China for many years. Electroacupuncture is the combination of an electrical stimulus and manual acupuncture, providing a therapy that can be specifically defined, with quantifiable intensity and frequency. Our previous study found that EA could attenuate amyloidosis and improve memory deficits in the APP/PS1 AD mouse model [30]. However, the mechanism of EA treatment on AD has not yet been comprehensively described. In this study, 7-month-old APP/PS1 double transgenic mice were selected as an animal model of AD. EA was stimulated at Baihui (GV20), Yintang (GV29), and Shuigou (GV26), all of which are located on governor vessel (GV). The present study examined the regulatory effects of repeated EA therapy on BACE1 expression and its associated substrates, to determine whether EA could effectively alleviate cognitive impairments of APP/PS1 mice and further elucidate the mechanism underlying the antidementia response of EA.

## 2. Materials and Methods

### 2.1. Animals and Grouping

Twenty 7-month-old male APP/PS1 mice and ten age- and gender-matched C57BL/6 mice were sourced from Beijing Hua Fukang Biotech (Certificate number: SCXK (Jing) 2014-0004), weighing 30 ± 2 g. The APP/PS1 mice were randomly divided into two groups, including the model (M) and electroacupuncture (EA) groups, ten mice in each group. The C57BL/6 mice served as the normal control (C) group. To avoid outside interference, all mice were housed separately in standard mouse cages under constant temperature (23 ± 2°C) and constant humidity (40-60%), with free access to water and food. The study was conducted in strict accordance with the Animal Ethics Committee of Beijing University of Chinese Medicine. We made every effort to minimize the suffering of the animals during the experimental procedure.

### 2.2. Electroacupuncture Treatment

**Figure 1:**
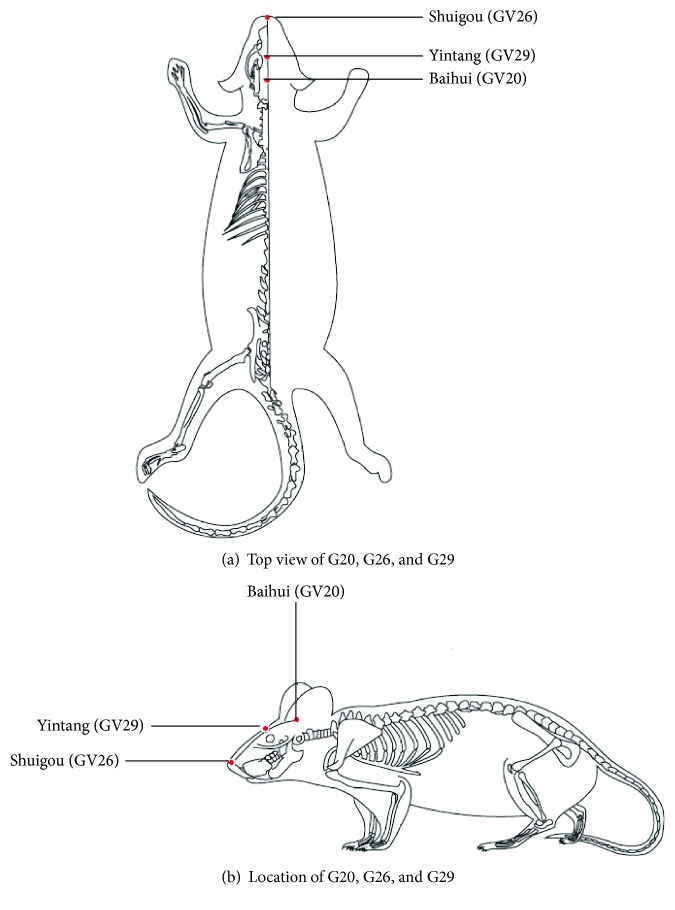
Locations of acupoints we applied in this study. Red points indicate the three governor vessel acupoints: Baihui (GV20), Yintang (GV29), and Shuigou (GV26).

Electroacupuncture was performed once every other day on the EA group. Prescription of acupuncture points included Baihui (GV20) (located on the bregma, midpoint of the linking line of mouse ears), Yintang (GV29) (located in the forehead, the middle depression of the two eyebrows medial end), and Shuigou (GV26) (located on the face, at the upper one-third of the philtrum) (Figure 1). Baihui (GV20) and Yintang (GV29) received EA intervention for 20 min, with intensity of 1 mA and frequency of 1 Hz, followed by fast pricking the point Shuigou (GV26). The treatment lasted for one month. No corresponding treatments were performed on the M and C groups, although the mice were grabbed and bound once every other day for one month to ensure an equivalent trial condition.

### 2.3. Morris Water Maze (MWM)

Cognitive impairment was assessed using the Morris water maze (MWM; XR-XM101, Xinruan, China). The MWM test included an acquisition trial and a probe test to measure memory. In the acquisition test (training trials), the location of the platform was fixed in the center of the third quadrant (target quadrant) throughout training and submerged approximately 1 cm beneath the water surface. Training trial was repeated for five consecutive days. The mice were gently released from the first, second, and fourth quadrants, respectively, in each trial, setting equal distance to the center of the tank, and a trial was terminated if the mouse failed to climb onto the platform within 60 seconds. Spatial acquisition performance was evaluated by assessing the escape latency to reach the platform. A 60-second probe trial was administered 24 h after the last training session. In the probe trial, the platform was removed from the pool and the mice were placed in the first, second, and fourth quadrants, respectively, and the swimming speed, frequency of crossing the platform in the target quadrant (the previous platform location), and percentage of time spent swimming within 60 s in the third quadrant housing the platform were derived. The data was measured by an automated analysis system (Daheng, China).

### 2.4. Immunofluorescence

The hippocampal sections were isolated and perfused with PBS and 0.2% Triton X-100 and then fixed in 4% paraformaldehyde for 24 h. The left brain sections were rapidly frozen under -50°C, followed by transversely cutting the tissue samples into 5 *μ*m slices. The slices were then incubated at 4°C with anti-APP (1 : 200, MAB348, Millipore, USA) and BACE1 (1 : 300, ab183612, Abcam, USA) antibodies. After permeabilization and blocking overnight, appropriate secondary antibodies (ab150113, ab150115, Abcam, USA) were used at a dilution of 1 : 200. After washing three times with PBS, the sections were incubated with DAPI (C0065, Solarbio, China) for 10 min, followed by live imaging. The hippocampal images were captured and obtained with a confocal laser microscope (FV1000, Olympus, Japan) at 1000x magnification.

### 2.5. Immunohistochemistry

Immunohistochemistry was performed on formalin-fixed, paraffin-embedded right brain hippocampal sections using three 5 *μ*m coronal hippocampal sections per mouse. Primary antibodies against APP (1 : 300, MAB348, Millipore, USA) and BACE1 (1 : 700, ab183612, Abcam, USA) were diluted in PBS, and the sections were incubated at 4°C overnight, washed, and then stained with biotinylated secondary antibody (KIT-9706, KIT-9701, MXB, China) for 10 min. For detection of the positive expression, the sections were stained with DAB (DAB2031, MXB, China) for 10 min. Microscopy (BX53, Olympus, China) was performed, and images were obtained at 100x and 400x magnification, respectively.

### 2.6. Western Blot (WB)

The extracted proteins were separated by electrophoresis with 10% SDS-PAGE. Gel was run at 80 V for 20 min and 120 V for 60 min and then transferred onto PVDF membranes at 4°C at 80 V for 1.5 h. The target proteins APP, BACE1, and PKA were measured by incubating with the primary antibodies against APP (1 : 2000, MAB348, Millipore, USA), BACE1 (1 : 2000, ab183612, Abcam, USA), p-PKA (1 : 2000, ab32390, Abcam, USA), and PKA (1 : 2000, ab75993, Abcam, USA) at 4°C overnight. After washing three times with TBST, the corresponding secondary antibody was used at a dilution of 1 : 2000 (bs-40295G, bs40296G, Bioss, China), followed by visualization with the ECL kit (mixed with 1 : 1, PE0010, Solarbio, China). The exposure was completed in a dark room with the chemiluminescence gel imaging system (C600, Azure Biosystems, USA). The antibodies against GAPDH (1 : 2000, TA-08, Zsbio, China) and *β*-actin (1 : 2000, bs-0061R, Bioss, China) were used as internal controls. Quantitative results were expressed as a ratio of APP to GAPDH, BACE1 to *β*-actin, and p-PKA to PKA and then compared in each group to measure relative changes.

### 2.7. Statistical Analysis

Statistical analysis was performed using the IBM SPSS Statistics 20 software. Comparison between groups was analyzed using ANOVA with the LSD post hoc test. Data was expressed as means ± SD ($\bar{x}$±*s*). Statistical significance was set at *p* < 0.05.

## 3. Results

### 3.1. Effect of EA Treatment on Cognitive Impairments of APP/PS1 Mice

APP/PS1 mice exhibited memory and learning impairments in the MWM test. EA treatment alleviated cognitive impairments in APP/PS1 mice.

#### 3.1.1. Training Trials

**Figure 2:**
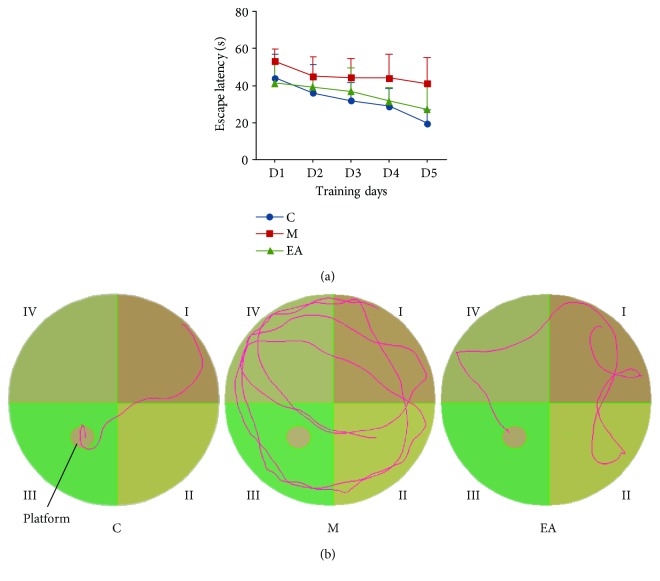
Training trial results of the MWM test after EA treatment. (a) Trends of escape latency of each group in training trials. (b) Representative training traces of each group on the fifth day (C: control; M: model; EA: electroacupuncture).

In the MWM training trials, spatial acquisition was assessed by the time spent by the mice in searching to climb onto the platform to escape the water (escape latency). The mice in each group presented a downward trend of escape latency with increase in training time. However, the M group showed worse acquisition performance over all training sessions, suggesting that the APP/PS1 dementia mice had obvious cognitive deficits (Table 1 and Figure 2(a)). Mice from the M group showed higher escape latency to reach the platform than the C group, which was significantly higher on days 3, 4, and 5. The EA treatment group showed evidently reduced escape latency than the M group on days 4 and 5 (Table 1 and Figure 2(a)).  Figure 2(b) showed representative training traces of each group on the fifth day.

#### 3.1.2. Probe Trial

**Figure 3:**
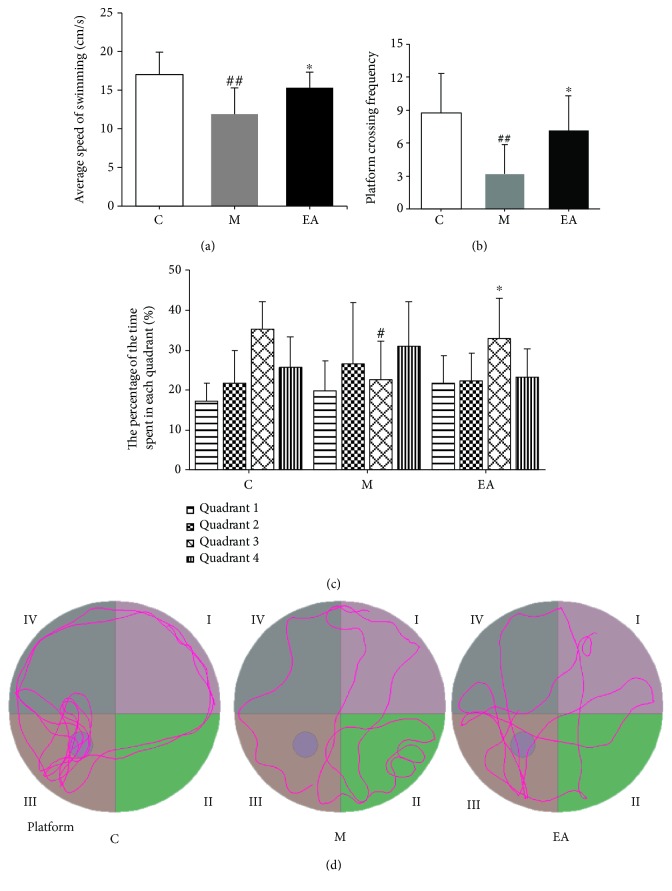
Probe trial results of the MWM test after EA treatment. (a) Average swimming speed of each group. (b) Platform crossing frequency of each group. (c) The proportion of time spent in each quadrant by each group. (d) Representative probe traces of each group. *n* = 10 per group. ^#^*p* < 0.05 vs. the C group, ^##^*p* < 0.01 vs. the C group, and ^∗^*p* < 0.05 vs. the M group (C: control; M: model; EA: electroacupuncture).

To assess the maintenance of memory, a probe trial was conducted on the training day 6. The M group presented a notably slower swimming speed as compared to the C group, and the velocity of the EA group was evidently higher than that of the M group (Table 2 and Figure 3(a)). Furthermore, the mice in the M group had significantly lesser frequency of platform crossing as compared to the C group. The EA treatment group showed higher frequency of crossing the platform than the M group (Table 2 and Figure 3(b)). The APP/PS1 mice spent less time in the target quadrant as compared to the C group. EA stimulation obviously improved memory performance as the EA group had higher retention in the third quadrant (Table 2 and Figure 3(c)). The above results demonstrated that the APP/PS1 double Tg mouse model had significant memory and learning disabilities, and EA intervention could ameliorate the cognitive deficits by improving spatial learning and memory.  Figure 3(d) showed representative probe traces of each group.

### 3.2. EA Treatment Decreased the Levels of BACE1 and APP in the Hippocampus of APP/PS1 Mice

**Figure 4:**
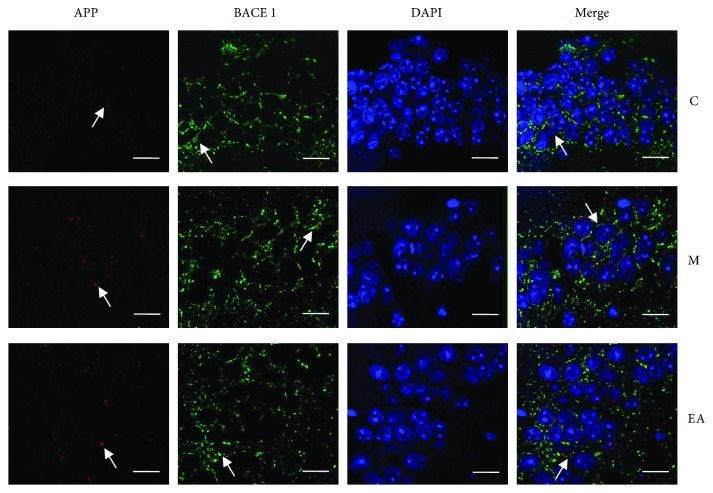
Effects of EA on coexpression of APP and BACE1 in the hippocampus of APP/PS1 mice using immunofluorescence. Scale bar = 20 *μ*m (C: control; M: model; EA: electroacupuncture). White arrows indicate the positive expressions of each protein.

**Figure 5:**
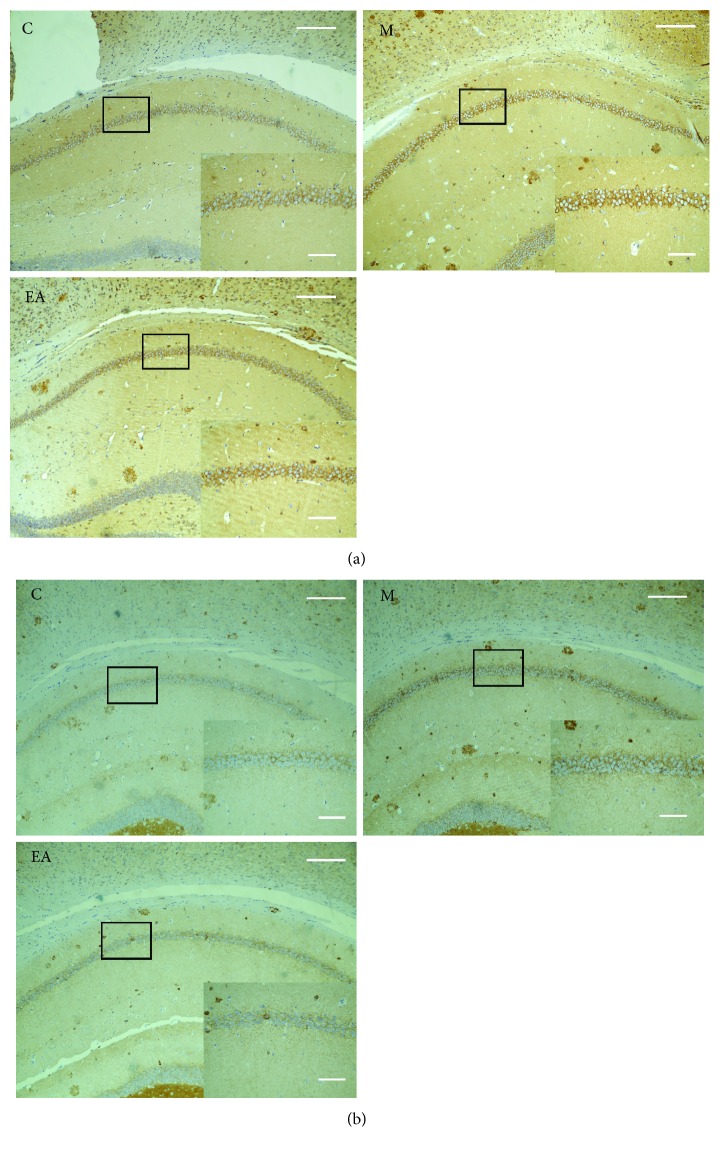
Effects of EA on expression levels of APP and BACE1 in the hippocampus of APP/PS1 mice in each group using immunohistochemistry. (a) The effects of EA treatment on the expression of hippocampal APP in APP/PS1 mice. (b) The effects of EA treatment on the expression of hippocampal BACE1 in APP/PS1 mice. Scale bar = 200 *μ*m or 50 *μ*m, in detail (C: control; M: model; EA: electroacupuncture).

**Figure 6:**
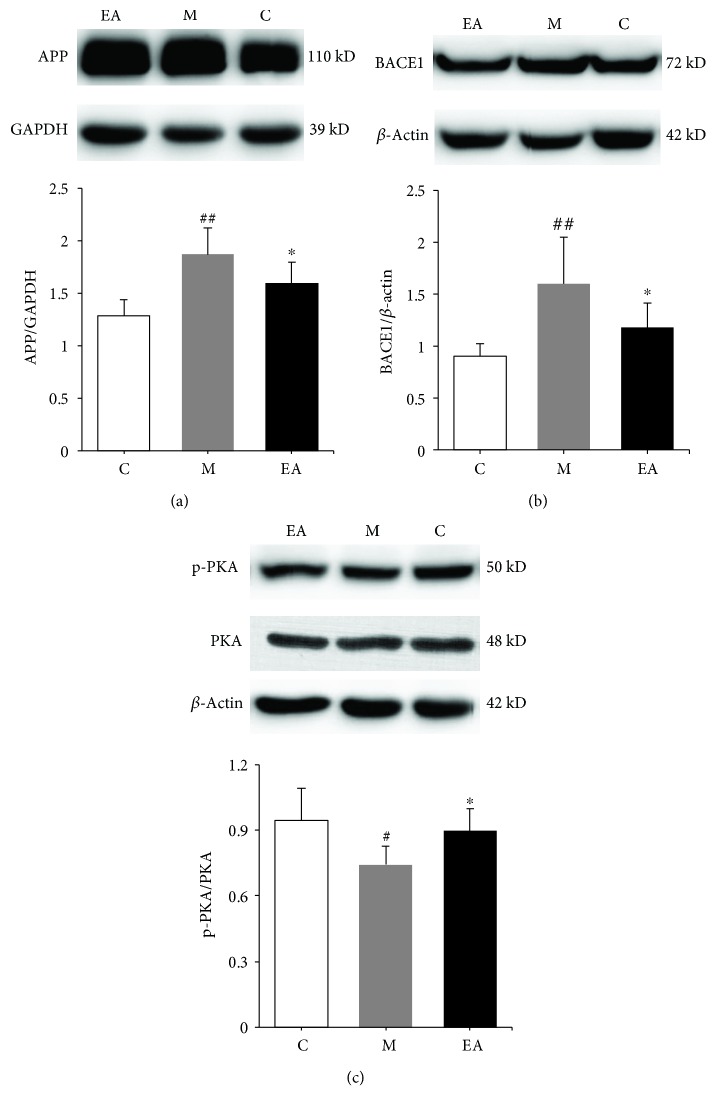
Effects of EA treatment on the expression levels of hippocampal APP, BACE1, and p-PKA in APP/PS1 mice using WB. (a) The effects of EA treatment on the relative expression of hippocampal APP in APP/PS1 mice. (b) The effects of EA treatment on the relative expression of hippocampal BACE1 in APP/PS1 mice. (c) The effects of EA treatment on the phosphorylation of PKA in the hippocampus of APP/PS1 mice. Data are presented as means ± SD; *n* = 7 per group. ^#^*p* < 0.05 vs. the C group, ^##^*p* < 0.01 vs. the C group, and ^∗^*p* < 0.05 vs. the M group (C: control; M: model; EA: electroacupuncture).

We next evaluated the distribution and accumulation of APP and BACE1 in mouse brain hippocampus by immunofluorescence, immunohistochemistry, and WB. BACE1 worked together with other enzymes to cleave APP into A*β* and acted as a rate-limiting enzyme. Immunofluorescence showed the coexpression of APP and BACE1 in the hippocampus (Figure 4). In the immunohistochemistry test, APP and BACE1 had obviously higher mean optical densities in the M group, which decreased after the EA treatment in the EA group (Figures 5(a) and 5(b)). Western blot results showed notably higher accumulation of APP and BACE1 in the M group as compared to the C group, while the EA group showed significantly lower expression of APP and BACE1 as compared to the M group (Figures 6(a) and 6(b)). These results confirmed the efficacy of EA stimulation in decreasing the deposition of APP and BACE1.

### 3.3. Effect of EA Treatment on the Activation of PKA in the Hippocampal Region of APP/PS1 Mice

We hypothesized that the decrease of BACE1 deposition might be accompanied by reduction of PKA activation. To test this possibility, we detected the phosphorylated level of PKA to further confirm the linkage between them. The phosphorylation and total level of hippocampal PKA was identified by western blot. As compared to the C group, the ratio of p-PKA/total-PKA in the M group was significantly lower. EA treatment upregulated the ratio of p-PKA/total-PKA (Figure 6(c)). The results suggested EA as a promising therapy to improve cognitive deficits by reversing the downphosphorylation of PKA.

## 4. Discussion

In this study, 7-month-old APP/PS1 double transgenic mice were used as an AD model. This specific mouse encodes a series of human AD genes and exhibits typical pathological symptoms of AD, which is well accepted in pathogenesis-related scientific studies [31–33]. Currently, the transgenic AD mouse model is widely adopted as a replacement of the traditional AD model, as it is more accurate, reliable, and effective [30–32]. In addition, these mice at approximately 7-8 months of age exhibited cerebral A*β* deposition [31, 34, 35] and local neuronal loss [36], followed by learning and memory deficits [31, 37]. The results of the MWM test showed that the escape latency of APP/PS1 AD mice was longer than the C group. In the probe test, mice in the M group showed worse memory maintenance, indicating that 7-month-old APP/PS1 mice were an appropriate and rational model for the present study.

Hippocampus is the fundamental region of the brain that is responsible for memory storage and is closely related to cognitive dysfunction. In the late 1960s, the hippocampus was reported to play a crucial role in LTP memory formation. Later, several studies confirmed that hippocampal atrophy is an extremely sensitive and specific indicator of early onset of amyloid plaque formation, excessive deposition of which could eventually lead to progression of AD [30, 32, 38]. Mouse model of AD with different gene mutation backgrounds was investigated to identify the region of the brain that is vulnerable to oxidative stress, and the hippocampus was more likely to be affected and damaged regardless of the genotype [38]. Importantly, the hippocampus is the principal focus of neuronal loss in AD, and the development of cognitive deficits in AD patients could be attributed to hippocampal damage, which is the first affected region in AD. Thus, hippocampus is of special interest for the therapeutic approach to neurodegenerative diseases.

Acupuncture originated in ancient China and is considered to play a homeostatic role in adjusting and maintaining the innate balance of *yin* and *yang*, through insertion and manipulation of needles over the body. EA is a modification of traditional Chinese acupuncture combined with modern electrotherapy, wherein electrical pulses continuously stimulate certain points during acupuncture. Several studies indicated that EA at certain points in AD mice could effectively protect against neuronal deterioration, and thus improve behavior and pathology assessment in neurodegenerative disease [31, 32, 37]. Over the last century, researches have tried to scientifically identify the anatomical structures of meridians and acupoints. Accumulative mechanistic discoveries reported that the regulatory responses of EA are directly induced by electrical stimulation of peripheral nerves or cutaneous nerves [39]. Under the stimulation of EA, the sensory nerve receptors are pathologically sensitized, which could trigger a series of a downstream dynamic process [40]. EA was found to be effective in the treatment of dementia [31, 32], since an objective and quantifiable electrical stimulus could optimize the effects of manual acupuncture. The theory of traditional Chinese medicine (TCM) believed that the pathogenesis of AD is closely correlated with the insufficiency of viscera, especially the deficiency of brain marrow, and phlegm stagnation-obstructed collaterals could worsen the brain functional abnormalities. In accordance with TCM theory, acupoints on governor vessel (GV) is the first choice in treating brain-related disease, since the circulation routine of GV is physically and pathologically linked to the brain through branches of meridians and collaterals. Therefore, we chose Baihui (GV 20), Yintang (GV 29), and Shuigou (GV 26), all of which are located on the GV pathway on the head as acupuncture points. The results of the MWM test demonstrated that repeated EA treatment could improve the cognitive deficits in APP/PS1 mice. The EA group showed shorter escape latency than the M group, faster swimming velocity, more crossing times of the previous platform, and occupied higher proportion in the target quadrant as compared to the M group, all of which were consistent with the previous reports [31, 37], suggesting that EA could serve as a potential therapy for AD.

AD is mainly characterized by A*β* accumulation in the brain, which is associated with pathogenesis like neurodegeneration, synaptic dysfunction, gliosis, and spatial disorientation [31–33, 41]. The imbalance between production and clearance of A*β* mainly contributes to the onset and deterioration of AD, especially soluble oligomers of A*β*_42_, the major metabolite of the APP and PS1 gene mutations [42], which could lead to synapse loss, inhibit LTP, and enhance long-term synaptic depression in the mouse hippocampus [43]. Genetic, biochemical, and behavioral research suggested that pathological generation of the neurotoxic A*β* peptide from sequential APP proteolysis is the crucial step in the development of AD [44]. BACE1, known as *β*-secretase, is highly expressed in multiple areas of neurons [13, 31, 45] and is especially enriched in axons [46]. The sequential cleavage of APP and numerous other proteolytic substrates mainly contributes to the generation of A*β* peptides and facilitates AD progression [13, 19]. In the present study, APP/PS1 mice showed higher expressions of APP and BACE1 in the hippocampus, which were dramatically downregulated by EA that exerted neuronal protection. This could be a mechanism involved in EA treatment for improving memory and learning abilities.

**Figure 7:**
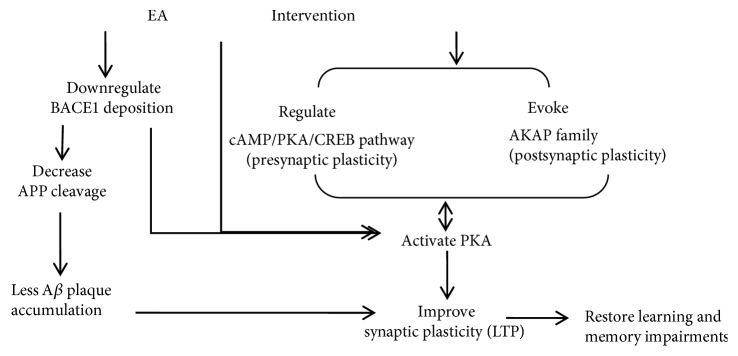
Schematic of the mechanism of EA intervention in ameliorating cognitive deficits in APP/PS1 double Tg mice.

To the best of our knowledge, many hypotheses suggested that the inhibition of BACE is fundamentally focused on decreasing the generation of A*β* peptides from the APP process, while BACE inhibitors could possess detrimental side effects on neuronal function. In this study, we observed that EA intervention on AD mice reduced BACE1 through downregulating APP cleavage to ameliorate cognitive impairments. Other substrates are also involved in improving memory and learning abilities of AD mice when stimulated with EA (Figure 7).

PKA, a crucial player in synaptic plasticity, was found in many rodent models [21, 25, 29]. Long-term synaptic plasticity has been intensively studied in the hippocampus [21, 26, 47]. Impaired hippocampal synaptic plasticity contributes to cognitive dysfunction in central neuronal diseases, such as AD [48]. In the present study, EA was found to upregulate PKA activation, further enhance synaptic plasticity, and improve memory in APP/PS1 mice. This study hypothesized that EA activated and phosphorylated PKA to help restore the impaired LTP of hippocampus-dependent memory. cAMP is an essential mediator that regulates various cell-specific processes, which directly activates the signaling protein PKA, while PKA acts as an upstream mediator of CREB. Typical PKA substrate RIM1*α* was involved in the cAMP/PKA signaling pathway, and Rim1*α* knockout mice exhibited severe cognitive deficits [49]. Using APP/PS1 double Tg mice combined with EA treatment could help elucidate the role of PKA in memory function. Our results suggested that the activation of PKA in the hippocampus may be potentially involved in the therapeutic effect of EA on AD.

Meanwhile, an increased level/activity of BACE1 was thought to be vital for pathogenesis of AD by modulating the activity of PKA in mouse brains [29]. Taken together, the overexpression of BACE1 may not only sequentially cleave APP to induce A*β* secretion but also inactivate the PKA pathway, which is independent of BACE1 enzymatic activity, leading to memory and learning deficits in the AD mouse model. All these findings suggested that BACE1 could be a disease-modifying therapeutic target to ameliorate cognitive impairments and alleviate multipathogenesis of APP/PS1 mice.

The results of this study strongly highlighted that EA could reverse cognitive dysfunction and enhance synaptic plasticity of AD mice, indicating the beneficial role of EA in neuron functional reconstruction. Regarding the downregulatory effects of EA on aberrant activation of BACE1, which noticeably reduced the amount of APP processing through the amyloidogenic pathway, we also found that EA directly or indirectly modulated the PKA signaling pathway to exert antidementia effects. However, the effects of EA on synaptic plasticity restoration need to be further explored. Moreover, additional research is needed to examine the PKA signaling pathway and validate the efficacy of EA, revealing the underlying mechanism of neuroprotection, in order to seek better strategies to prevent and alleviate AD in clinical trials.

## 5. Conclusions

The behavioral test and protein detection results in this study showed that EA could downregulate APP and BACE1 expression and activate the PKA pathway to ameliorate memory dysfunction. These results provided preliminary proof for EA treatment as a complementary replacement for AD pathogenesis.

## Figures and Tables

**Table 1:** Comparison of escape latency time of each group in training trials.

Group	*n*	Day 1	Day 2	Day 3	Day 4	Day 5
C	10	44.15 ± 12.97	35.93 ± 15.45	28.35 ± 11.33	24.63 ± 10.52	19.57 ± 7.34
M	10	53.04 ± 6.69	42.65 ± 11.61	42.42 ± 11.06^#^	40.52 ± 16.15^#^	41.13 ± 14.17^##^
EA	10	41.43 ± 10.36	39.25 ± 6.26	36.88 ± 12.72	34.87 ± 8.02^∗^	27.13 ± 13.28^∗^

Results are presented as means ± SD; *n* = 10 per group. ^#^*p* < 0.05 vs. the C group, ^##^*p* < 0.01 vs. the C group, and ^∗^*p* < 0.05 vs. the M group (C: control; M: model; EA: electroacupuncture).

**Table 2:** Comparison of memory maintenance of each group in probe trial.

Group	*n*	Average speed of swimming (cm/s)	Platform crossing frequency	The percentage of time spent in the target quadrant (%)
C	10	17.02 ± 2.90	8.78 ± 3.53	0.35 ± 0.07
M	10	11.91 ± 3.38^##^	3.17 ± 2.71^##^	0.23 ± 0.10^#^
EA	10	15.30 ± 2.03^∗^	7.14 ± 3.18^∗^	0.33 ± 0.10^∗^

Results are presented as means ± SD; *n* = 10 per group. ^#^*p* < 0.05 vs. the C group, ^##^*p* < 0.01 vs. the C group, and ^∗^*p* < 0.05 vs. the M group (C: control; M: model; EA: electroacupuncture).

## Data Availability

The data used to support the findings of this study are available from the corresponding author upon reasonable request.
